# Contrasting change in biomass translocation with environment in two rice hybrids

**DOI:** 10.1371/journal.pone.0220651

**Published:** 2019-07-31

**Authors:** Min Huang, Zui Tao, Tao Lei, Jiana Chen, Fangbo Cao, Xiaohong Yin, Yingbin Zou

**Affiliations:** 1 Crop and Environment Research Center, College of Agronomy, Hunan Agricultural University, Changsha, China; 2 Guangxi Key Laboratory of Rice Genetics and Breeding, Rice Research Institute, Guangxi Academy of Agricultural Sciences, Nanning, China; ICAR-Indian Institute of Rice Research, INDIA

## Abstract

Translocation of biomass produced during pre-heading to grains is a determinant of grain yield, but also plays an important role in adaptation to unfavorable environments during post-heading in rice. In this study, field experiments were conducted to determine the critical factors that regulate biomass translocation in rice. Biomass translocation and production characteristics of two rice hybrids (Guiliangyou 2 and Y-liangyou 1) were compared between two site-year environments (Naning-2014 and Yongan-2018). Results showed that biomass translocation parameters (biomass translocation amount and rate and contribution of biomass translocation to filled grain weight) and ratio of biomass production during pre-heading to post-heading (BP_pre_/BP_post_ ratio) decreased in Guiliangyou 2 but increased in Y-liangyou 1 with the environment change from Nanning-2014 to Yongan-2018. The decreased BP_pre_/BP_post_ in Guiliangyou 2 was attributable to increased biomass production during post-heading (BP_post_), while the increased BP_pre_/BP_post_ ratio in Y-liangyou 1 was due to increased biomass production during pre-heading (BP_pre_). Higher cumulative incident solar radiation and larger diurnal temperature variation were responsible for the increased BP_post_ in Guiliangyou 2 and the increased BP_pre_ in Y-liangyou 1 grown in Yongan in 2018 compared to in Nanning in 2014. The results of this study indicate that changes in biomass translocation and production with environment (climate) in rice are dependent on genotype and that the BP_pre_/BP_post_ ratio is an important factor regulating biomass translocation in rice.

## Introduction

Rice is important for world food security in that it is the staple food for more than half of the global population [1]. To produce enough rice to meet the growing demand for food resulting from population growth and economic development, improving yield has been the first priority in rice breeding and production for a long time [2, 3]. As a consequence of the development of new varieties such as semi-dwarf cultivars in the late 1950s and hybrid rice cultivars in the late 1970s as well as the improvement of crop management practices such as fertilization and irrigation, rice yield has more than doubled in most parts of the world and even tripled in certain countries such as China over the past several decades [4, 5].

Although increasing yield has been, and probably will remain, the chief objective in rice production, improving yield stability (i.e., the ability of a crop to maintain yield performance across diverse environments) is also a primary objective [6, 7, 8]. This objective has become more and more important because of climate change, which may result in increased intensity and frequency of extreme weather events in the future [9, 10, 11].

In rice crops, translocation of biomass produced during pre-heading to grains influences grain yield under favorable conditions [12], but also plays an important role in adapting to sub-optimum or stress environments, such as high temperature and water stress, during post-heading [13, 14, 15]. A greater fundamental understanding of the characteristics of biomass translocation would be useful for providing information on achieving high and stable grain yield in rice.

Studies in rice have shown that biomass translocation can largely vary with genotype and environment [12, 16, 17, 18]. However, limited information is available on the factors that are responsible for variation in biomass translocation. In the present study, we compared climatic factors and characteristics of biomass translocation and production in two rice hybrids in two environments. Our objective was to determine the critical factors that regulate biomass translocation in rice.

## Materials and methods

### Ethics statements

No specific permissions were required for the activities conducted in this study. The field used in this study is neither privately owned nor protected. The experiments did not involve endangered or protected species.

### Experimental details

Field experiments were conducted in Nanning (22°51′ N, 108°17′ E, 78 m asl), Guangxi Province, China in the early rice-growing season (from March to July) in 2014 and in Yongan (28°09′ N, 113°37′ E, 43 m asl), Hunan Province, China in the single rice-growing season (from May to September) in 2018. The soil of the experiment field was clay in texture in both sites. The soil chemical properties at the upper 20 cm layer were: pH 6.33, 24.1 g organic matter kg^–1^, 142 mg available N kg^–1^, 34.8 mg available P kg^–1^, and 123 mg available K kg^–1^ in Nanning; and pH 6.16, 34.8 g organic matter kg^–1^, 140 mg available N kg^–1^, 28.9 mg available P kg^–1^, and 118 mg available K kg^–1^ in Yongan.

Two rice hybrids, Guiliangyou 2 and Y-liangyou 1, were used in the experiment. Guiliangyou 2 and Y-liangyou 1 are two-line hybrids released in Guangxi Province in 2008 and in Hunan Province in 2006, respectively. These two hybrids were selected because: (1) they are used in different provinces and hence may have different responses to environmental change; and (2) they have been widely grown by local rice farmers due to their high yield potential. The two hybrids were arranged in a randomized complete-block design with three replications in Nanning and four replications in Yongan. The plot size was 25 m^2^ in Nanning and 60 m^2^ in Yongan.

Pre-germinated seeds were sown on 8 March in Nanning in 2014 and on 5 May in Yongan in 2018. Twenty five- and 20-day-old seedlings were transplanted in Nanning and Yongan, respectively. Transplanting was done at a hill spacing of 20 cm × 20 cm with two seedlings per hill. Crops were managed according to local recommended practices. In Nanning, the plots received 165 kg N ha^–1^, 54 kg P_2_O_5_ ha^–1^, and 180 kg K_2_O ha^–1^. N and K were applied in three splits: 50% as basal fertilizer (1 day before transplanting), 30% at early-tillering (7 days after transplanting), and 20% at panicle initiation. P was applied as basal fertilizer. In Yongan, the plots received 150 kg N ha^–1^, 75 kg P_2_O_5_ ha^–1^, and 150 kg K_2_O ha^–1^. N was applied in three splits: 50% as basal fertilizer, 30% at early-tillering, and 20% at panicle initiation. P was applied as basal fertilizer. K was split equally as basal fertilizer and at panicle initiation. The experimental field was kept flooded from transplanting until 7 days before maturity in both sites. Insects, diseases, and weeds were intensively controlled by chemicals.

**Fig 1:**
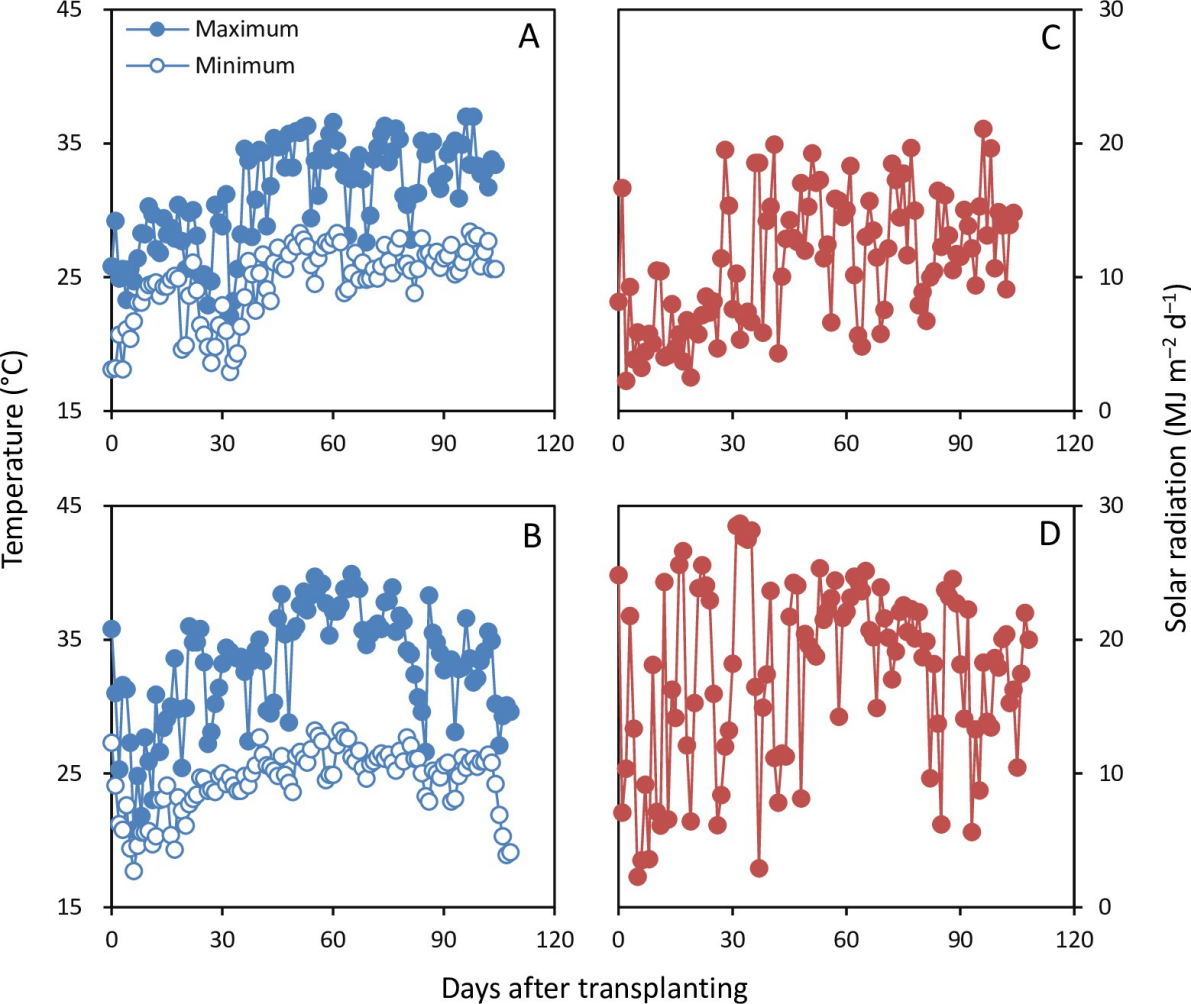
Daily maximum and minimum temperatures (A and B) and solar radiation (C and D) during the rice-growing season in Nanning in 2014 (A and C) and Yongan (B and D) in 2018.

Daily maximum and minimum temperatures and incident solar radiation during the rice-growing season were recorded using an automatic weather station (Vantage Pro2, Davis Instruments Corp., Hayward, CA, USA) (Fig 1A–1D). Twelve hills were sampled from each plot at heading and maturity. Plant samples were separated into leaves, stems, and panicles at heading and into straw and filled and unfilled grains at maturity. Dry weight of each plant organ was determined after oven-drying at 70°C to a constant weight. Biomass production during pre-heading (BP_pre_) was the summation of dry weights of leaves, stems, and panicles at heading.

Biomass production during post-heading (BP_post_) was calculated by subtracting BP_pre_ from the total biomass at maturity (i.e., the summation of dry weights of straw and filled and unfilled grains at maturity). The BP_pre_/BP_post_ ratio was calculated by dividing BP_pre_ by BP_post_. Biomass translocation amount (BT_a_) was calculated according to Yang et al. [12]. Biomass translocation rate (BT_r_) was calculated as the percentage of BT_a_ divided by BP_pre_. Contribution of biomass translocation to filled grain weight (BT_c_) was calculated as the percentage of BT_a_ divided by the dry weight of filled grains at maturity. Apparent radiation use efficiency during pre-heading (RUE_pre_) and post-heading (RUE_post_) were calculated by dividing BP_pre_ and BP_post_ by the cumulative incident solar radiation during pre-heading and post-heading, respectively. The RUE_pre_/RUE_post_ ratio was calculated by dividing RUE_pre_ by RUE_post_.

### Statistical analysis

Data were subjected to analysis of variance using Statistix 8.0 software (Tallahassee, FL, USA). Means were compared based on the least significant difference test (LSD) at the 0.05 probability level.

## Results

Pre-heading duration was five and four days longer in Nanning in 2014 than in Yongan in 2018 for Guiliangyou 2 and Y-liangyou 1, respectively (Table 1). Post-heading duration was ten and eight days shorter in Nanning in 2014 than in Yongan in 2018 for Guiliangyou 2 and Y-liangyou 1, respectively. Average daily maximum temperature during pre-heading in Nanning in 2014 was 2.1°C and 2.5°C lower than that in Yongan in 2018 for Guiliangyou 2 and Y-liangyou 1, respectively. Average daily maximum temperature during post-heading was 1.7°C lower in Nanning in 2014 than in Yongan in 2018 for Guiliangyou 2, whereas it was 0.1°C higher in Nanning in 2014 than in Yongan in 2018 for Y-liangyou 1. Average daily minimum temperature during pre-heading in Nanning in 2014 was 0.2°C and 0.3°C lower than that in Yongan in 2018 for Guiliangyou 2 and Y-liangyou 1, respectively. Average daily minimum temperature during post-heading was 0.6°C higher in Nanning in 2014 than in Yongan in 2018 for both Guiliangyou 2 and Y-liangyou 1. Cumulative incident solar radiation during pre-heading was 36% and 37% lower in Nanning in 2014 than in Yongan in 2018 for Guiliangyou 2 and Y-liangyou 1, respectively. Cumulative incident solar radiation during post-heading in Nanning in 2014 was 50% and 42% lower than that in Yongan in 2018 for Guiliangyou 2 and Y-liangyou 1, respectively.

**Table 1:** Growth duration, average daily maximum and minimum temperatures, and cumulative incident solar radiation during pre- and post-heading of two rice hybrids grown in Nanning in 2014 and Yongan in 2018.

Rice hybrid	Site-year	Growth duration (d)		Daily maximum temperature (°C)		Daily minimum temperature (°C)		Incident solar radiation (MJ m^−2^)	
		Pre-heading	Post-heading	Pre-heading	Post-heading	Pre-heading	Post-heading	Pre-heading	Post-heading
Guiliangyou 2	Naning-2014	67	28	30.2	33.1	23.8	26.1	693	353
	Yongan-2018	62	38	32.3	34.8	24.0	25.7	1081	705
Y-liangyou 1	Naning-2014	76	28	30.5	33.4	24.0	26.5	810	367
	Yongan-2018	72	36	33.0	33.3	24.3	24.9	1292	635

**Fig 2:**
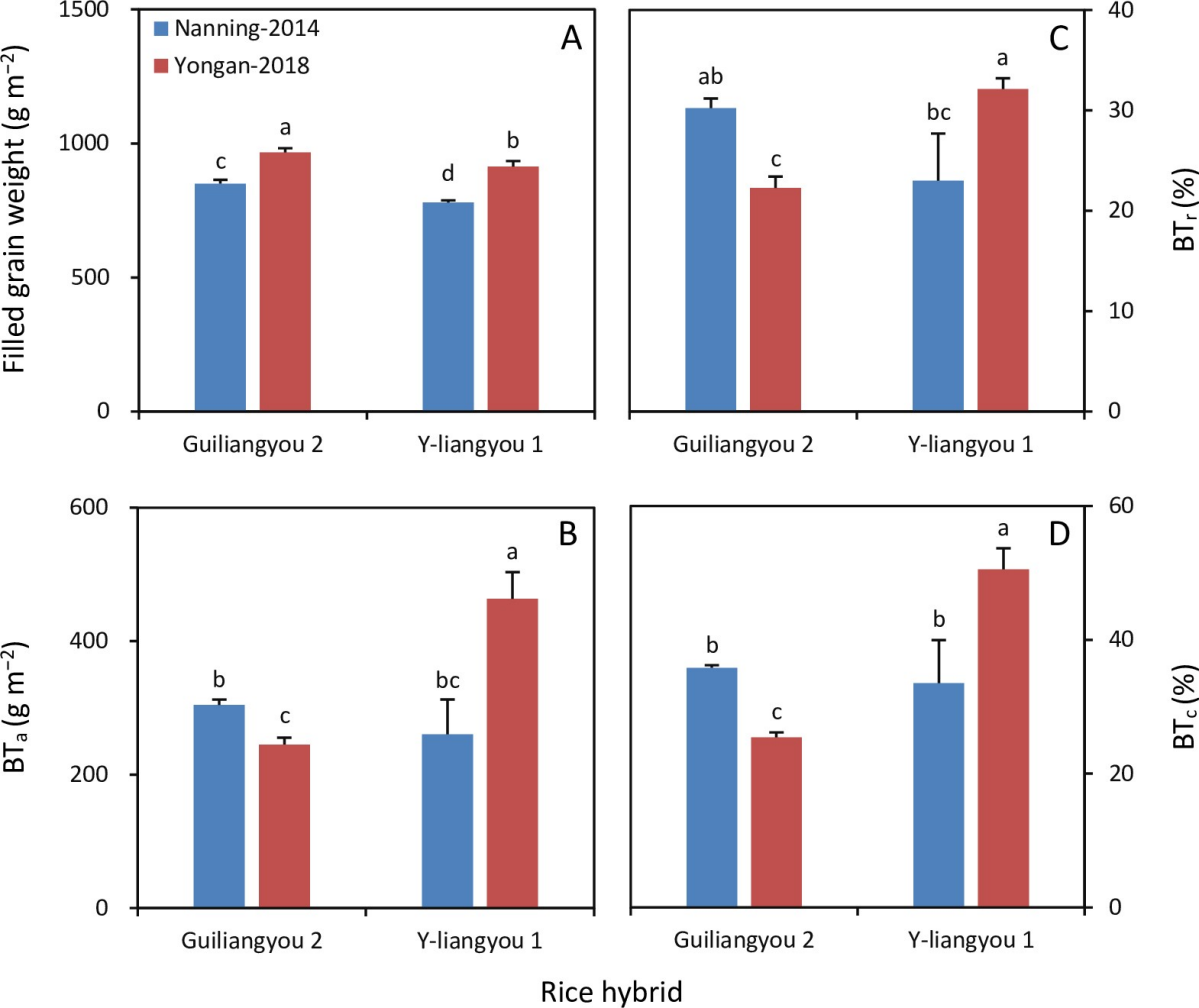
**Filled grain weight (A), biomass translocation amount (BT**_**a**_**, B) and rate (BT**_**r**_**, C), and contribution of biomass translocation to filled grain weight (BT**_**c**_**, D) in two rice hybrids grown in Nanning in 2014 and Yongan in 2018.** Error bars are SE. Within a subfigure, bars not sharing any letter are significantly different by the LSD test at the 0.05 probability level.

Filled grain weight in Nanning in 2014 was 12% and 15% lower than that in Yongan in 2018 for Guiliangyou 2 and Y-liangyou 1, respectively (Fig 2A). BT_a_ was 24% higher in Nanning in 2014 than in Yongan in 2018 for Guiliangyou 2, while it was 44% lower in Nanning in 2014 than in Yongan in 2018 for Y-liangyou 1 (Fig 2B). BT_r_ was 8% higher in Nanning in 2014 than in Yongan in 2018 for Guiliangyou 2, whereas it was 9% lower in Nanning in 2014 than in Yongan in 2018 for Y-liangyou 1 (Fig 2C). BT_c_ was 10% higher in Nanning in 2014 than in Yongan in 2018 for Guiliangyou 2, while it was 17% lower in Nanning in 2014 than in Yongan in 2018 for Y-liangyou 1 (Fig 2D).

**Fig 3:**
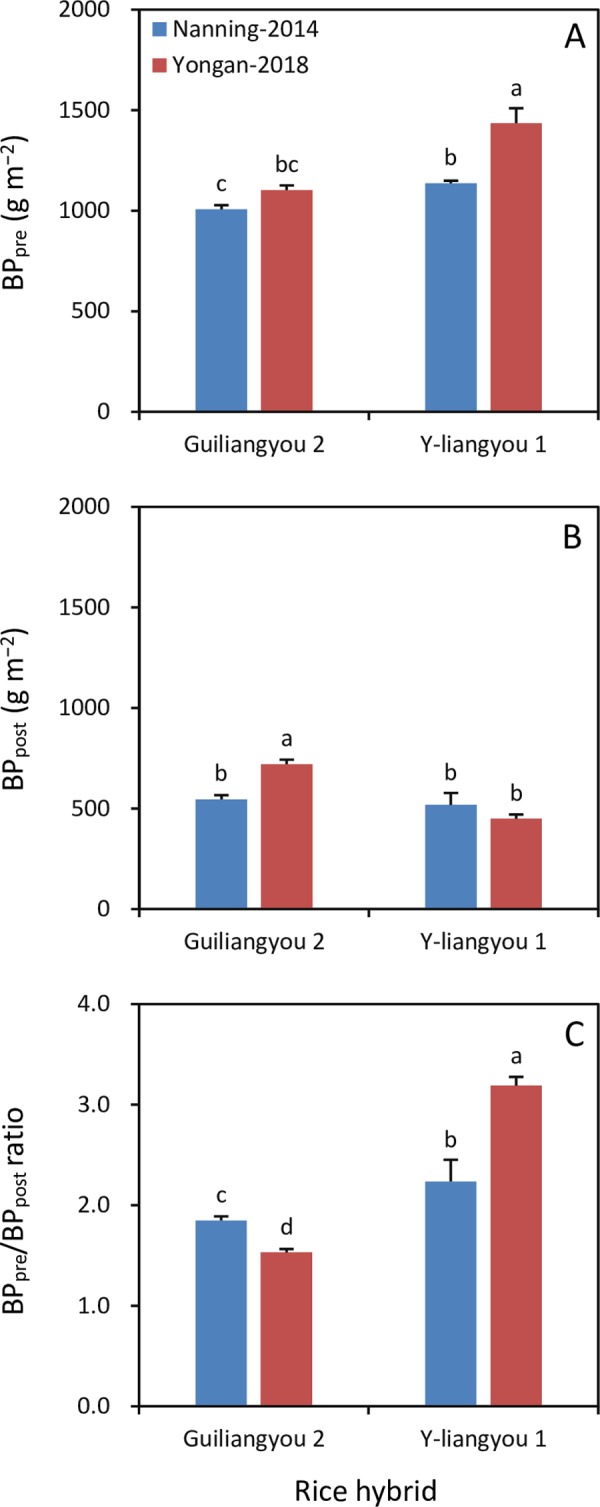
**Biomass production during pre-heading (BP**_**pre**_**, A) and post-heading (BP**_**post**_**, B) and BP**_**pre**_**/BP**_**post**_ **ratio (C) in two rice hybrids grown in Nanning in 2014 and Yongan in 2018.** Error bars are SE. Within a subfigure, bars not sharing any letter are significantly different by the LSD test at the 0.05 probability level.

There was no significant difference in BP_pre_ between the two environments for Guiliangyou 2, whereas for Y-liangyou 1 it was 21% lower in Nanning in 2014 than in Yongan in 2018 (Fig 3A). BP_post_ in Nanning in 2014 was 24% lower than that in Yongan in 2018 for Guiliangyou 2, while the difference was not significant for Y-liangyou 1 (Fig 3B). The BP_pre_/BP_post_ ratio was 21% higher in Nanning in 2014 than in Yongan in 2018 for Guiliangyou 2, whereas it was 30% lower in Nanning in 2014 than in Yongan in 2018 for Y-liangyou 1 (Fig 3C).

**Fig 4:**
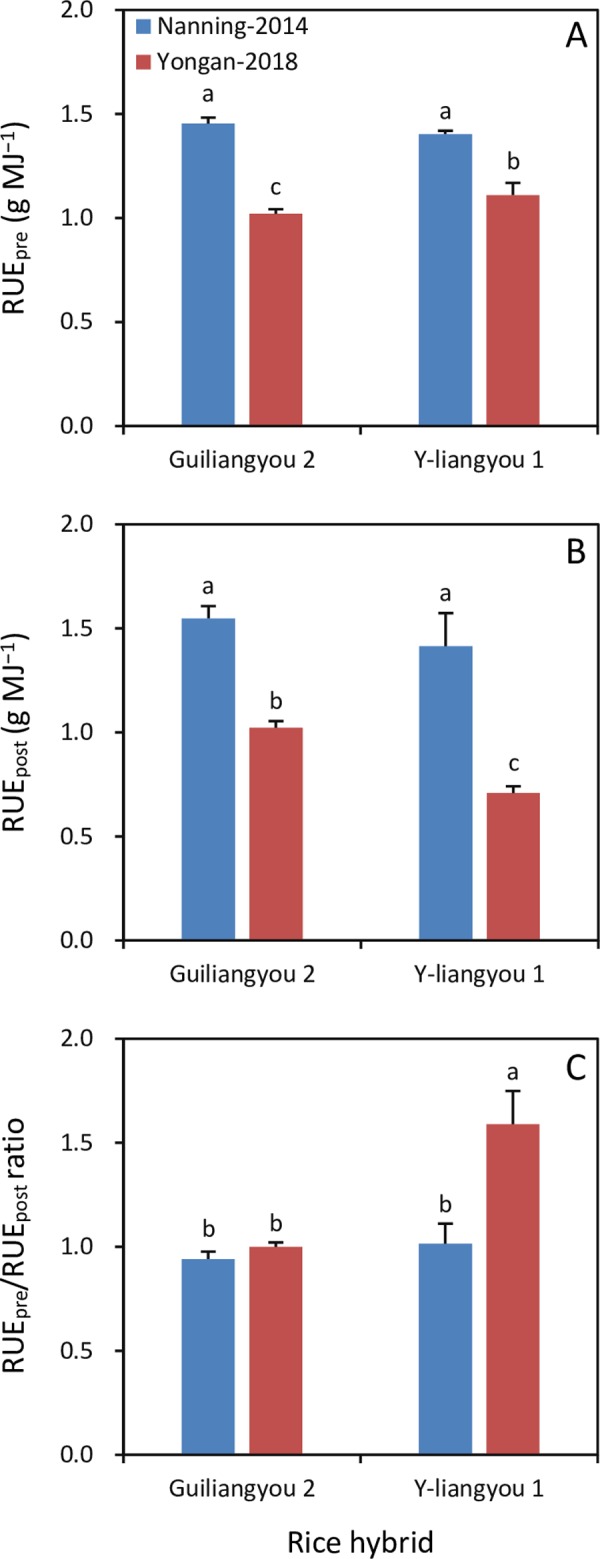
**Apparent radiation use efficiency during pre-heading (RUE**_**pre**_**, A) and post-heading (RUE**_**post**_**, B) and RUE**_**pre**_**/RUE**_**post**_ **ratio (C) in two rice hybrids grown in Nanning in 2014 and Yongan in 2018.** Error bars are SE. Within a subfigure, bars not sharing any letter are significantly different by the LSD test at the 0.05 probability level.

RUE_pre_ was 42% and 26% higher in Nanning in 2014 than in Yongan in 2018 for Guiliangyou 2 and Y-liangyou 1, respectively (Fig 4A). RUE_post_ in Nanning in 2014 was 51% and 100% higher than that in Yongan in 2018 for Guiliangyou 2 and Y-liangyou 1, respectively (Fig 4B). The difference in RUE_pre_/RUE_post_ ratio was not significant between the two environments for Guiliangyou 2, while it was 36% lower in Nanning in 2014 than in Yongan in 2018 for Y-liangyou 1 (Fig 4C).

## Discussion

In this study, considerable differences in climatic conditions including temperature and solar radiation existed between the two tested rice-growing environments; the differences are consistent with those observed in adjacent regions in previous studies [18, 19]. Although the two tested rice hybrids had similar responses to the environmental variation in term of filled grain weight, their responses for biomass translocation were contrasting. This finding indicates that there may not be a single relationship between grain yield and biomass translocation in rice. This may also be why contradictory results have been reported in previous studies: Weng et al. [16] and Miah et al. [17] observed that high-yielding rice had higher BT_a_, while Yang et al. [12] reported that higher grain yield was not associated with BT_a_ in rice.

Prior to the present study, few studies had investigated the factors that contribute to the variation in biomass translocation across genotypes and environments. The results of this study showed that the changes in the biomass translocation parameters (BT_a_, BT_r_, and BT_c_) with environment were similar to that for the BP_pre_/BP_post_ ratio. Namely, BT_a_, BT_r_, BT_c_, and the BP_pre_/BP_post_ ratio decreased in Guiliangyou 2 but increased in Y-liangyou 1 with the environment change from Nanning-2014 to Yongan-2018. This result demonstrates that the BP_pre_/BP_post_ ratio is a critical factor regulating biomass translocation in rice. This finding can also explain why increased biomass translocation occurred synchronously with early plant senescence and reduced photosynthesis in rice grown under water stress during post-heading [14]. These facts suggest that crop self-adjustment to environmental changes plays an important role in achieving stable grain yield in rice and highlight the need for a fundamental understanding of the self-adjustment mechanisms.

Biomass production is determined by environmental factors including climatic variables and plant traits such as radiation use efficiency in rice crops, and the latter varies with genotype and environment [20, 21, 22]. In this study, the decreased BP_pre_/BP_post_ ratio following the environment change from Nanning-2014 to Yongan-2018 in Guiliangyou 2 was attributable to increased BP_post_, because the change in BP_pre_ was not significant. Higher cumulative incident solar radiation and larger diurnal temperature variation (i.e., the difference between maximum and minimum temperatures) during post-heading were responsible for the increased BP_post_ in Guiliangyou 2 grown in Yongan in 2018 than in Nanning in 2014. On the contrary, the increased BP_pre_/BP_post_ ratio with the environment change from Nanning-2014 to Yongan-2018 in Y-liangyou 1 was due to increased BP_pre_, because there was no significant change in BP_post_. The increased BP_pre_ in Y-liangyou 1 grown in Yongan in 2018 compared to that grown in Nanning in 2014 was attributable to higher cumulative incident solar radiation and larger diurnal temperature variation during post-heading.

The contrasting changes in BP_pre_ with environment in Guiliangyou 2 and Y-liangyou 1 indicate that these two rice hybrids have different sensitivities to climatic variation during pre-heading, namely, Y-liangyou 1 is more sensitive than Guiliangyou 2. This difference was also partly responsible for the contrasting changes in BP_post_ with environment in the two rice hybrids. For Y-liangyou 1, its sensitivity to the environmental change from Nanning-2014 to Yongan-2018 resulted in a considerable increase in BP_pre_, which could lead to a high population density and consequently an increase in mutual shading of plants, an acceleration in leaf senescence, and a reduction in photosynthetic capacity [23]. This outcome could be supported by the finding that the RUE_pre_/RUE_post_ ratio significantly increased with environmental change from Nanning-2014 to Yongan-2018 for Y-liangyou 1. In contrast, these outcomes were not observed in the insensitive rice hybrid Guiliangyou 2.

## References

[1] Muthayya S, Sugimoto JD, Montgomery S, Maberly GF. An overview of global rice production, supply, trade, and consumption. Ann NY Acad Sci. 2014; 1324: 7–14. 10.1111/nyas.12540 25224455

[2] Normile D. Reinventing rice to feed the world. Science. 2008; 321: 330–333. 10.1126/science.321.5887.330 18635770

[3] Peng S, Khush GS, Virk P, Tang Q, Zou Y. Progress in ideotype breeding to increase rice yield potential. Field Crops Res. 2008; 108: 32–38.

[4] Peng S, Zou Y, Tang Q. Current status and challenges of rice production in China. Plant Prod Sci. 2009; 12: 3–8.

[5] Zhang Q. Strategies for developing green super rice. Proc Natl Acad Sci USA. 2007; 104: 16402–16409. 10.1073/pnas.0708013104 17923667 PMC2034246

[6] Calderini DF, Slafer GA. Changes in yield and yield stability in wheat during the 20th century. Field Crops Res. 1998; 57: 335–347.

[7] Rakshit S, Hariprasanna K, Gomashe S, Ganapathy KN, Das IK, Ramana OV, et al Changes in area, yield gains, and yield stability of sorghum in major sorghum-producing countries, 1970 to 2009. Crop Sci. 2014; 54: 1571–1584.

[8] Yin X, Huang M, Zou Y. Changes in rice yield stability in southern China from 1949 to 2015. Agric Environ Lett. 2018; 3: 170038.

[9] Rosenzweig C, Iglesias A, Yang XB, Epstein PR, Chivian E. Climate change and extreme weather events: Implications for food production, plant diseases, and pests. Glob Change Human Health. 2001; 2: 90–104.

[10] Qiu J. China drought highlights future climate threats. Nature. 2010; 465: 142–143. 10.1038/465142a 20463708

[11] Zhang Z, Wang P, Chen Y, Song X, Wei X, Shi P. Global warming over 1960–2009 did increase heat stress and reduce cold stress in the major rice-planting areas across China. Eur J Agron. 2014; 59: 49–56.

[12] Yang W, Peng S, Laza RC, Visperas RM, Dionisio-Sese ML. Yield gap analysis between dry and wet season rice crop grown under high-yielding management conditions. Agron J. 2008; 100: 1390–1395.

[13] Laza RC, Peng S, Akita S, Saka H. Contribution of biomass partitioning and translocation of grain yield under sub-optimum growing conditions in irrigated rice. Plant Prod Sci. 2003; 6: 28–35.

[14] Yang J, Zhang J. Grain filling of cereals under soil drying. New Phytol. 2006; 169: 223–236. 10.1111/j.1469-8137.2005.01597.x 16411926

[15] Shi W, Muthurajan R, Rahman H, Selvam J, Peng S, Zou Y, et al Source-sink dynamics and proteomic reprogramming under elevated night temperature and their impact on rice yield and grain quality. New Phytol. 2013; 197: 825–837. 10.1111/nph.12088 23252708

[16] Weng J, Takeda T, Agata W, Hakoyama S. Studies on dry matter and grain production of rice plants: I. Influence of reserved carbohydrate until heading stage and assimilation products during ripening period on grain production. Jpn J Crop Sci. 1982; 51: 500–509.

[17] Miah MNH, Yoshida T, Yamamoto Y, Nitta Y. Characteristics of dry matter production and partitioning of dry matter in high yielding semi-dwarf indica and japonica-indica hybrid rice varieties. Jpn J Crop Sci. 1996; 65: 672–685.

[18] Huang M, Zhang R, Jiang P, Xie X, Zhou X, Cao F, et al Temperature-related yield constraints of early-rice in south China: A cross-location analysis. PLoS ONE. 2016; 11: e0158601 10.1371/journal.pone.0158601 27366908 PMC4930191

[19] Jiang P, Xie X, Huang M, Zhou X, Zhang R, Chen J, et al Potential yield increase of hybrid rice at five locations in southern China. Rice. 2016; 9: 11 10.1186/s12284-016-0085-6 26984118 PMC4794477

[20] Ying J, Peng S, He Q, Yang H, Yang C, Visperas RM, et al Comparison of high-yield rice in tropical and subtropical environments I. Determinants of grain and dry matter yields. Field Crops Res. 1998; 57: 71–84

[21] Huang M, Shan S, Zhou X, Chen J, Cao F, Jiang L, et al Leaf photosynthetic performance related to higher radiation use efficiency and grain yield in hybrid rice. Field Crops Res. 2016; 193: 87–93.

[22] Huang M, Shan S, Cao F, Zou Y. The solar radiation-related determinants of rice yield variation across a wide range of regions. NJAS Wagen J Life Sci. 2016; 78: 123–128.

[23] Huang M, Zou Y, Jiang P, Xia B, Feng Y, Cheng Z, et al Effect of tillage on soil and crop properties of wet-seeded flooded rice. Field Crops Res. 2012; 129: 28–38.

